# Antibiotic resistance patterns of *Staphylococcus aureus* and Enterobacteriaceae isolated from street foods in selected towns of Ethiopia

**DOI:** 10.1186/s12879-024-09266-4

**Published:** 2024-04-02

**Authors:** Mathewos Moges, Ernst Kristian Rodland, Tesfaye Legesse, Ambelu Argaw

**Affiliations:** 1 Environmental Health Science and Technology Department, Jimma University, Jimma, Ethiopia; 2 Division of Water and Health, Ethiopian Institute of Water Resources, Addis Abeba University, Addis Abeba, Ethiopia; 3 Norwegian Institute of Public Health, Oslo, Norway; 4 Ethiopian Public Health Institute, Addis Abeba, Ethiopia

**Keywords:** Street food, Ready-to-eat foods, Antibiotic resistance, Antibiotic sensitivity

## Abstract

**Background:**

Street foods are any foods or drinks prepared or sold by street vendors in an open space. The purpose of this study was to determine the Bacteriological safety and antibiotic resistance patterns of *Staphylococcus aureus* and Enterobacteriaceae isolated from street foods.

**Method:**

A laboratory-based cross-sectional study was used from December 2022 to February 2023 on street foods of Addis Ababa, Hawassa, Dire Dawa, and Jimma towns of Ethiopia. 525 street foods and 175 water samples were taken from 175 street food vending stalls. Proportional allocation to the total town population and stratified sampling techniques were used to select vending stalls. Samples were analyzed for the presence of bacteria following the standard microbiological methods used for the isolation, enumeration, and identification of bacteria. Pour plate technique was used to transfer the suspension to MacConkey agar, Mannitol Salt Agar, and Salmonella Shigella Agar. The antibiotic susceptibility test was performed using the Kirby-Bauer disk diffusion method. SPSS software was used to analyze the data.

**Result:**

Out of 525 food samples, 279 (53%) were contaminated by bacteria. From 175 water samples, 95 (54.3%) were contaminated with *Escherichia coli*. From both samples in total, eleven bacterial species were isolated. *Staphylococcus aureus* was the most frequently isolated species. *Shigella*, *Klebsiella*, and *Salmonella group A* have statistically significant with the type of food. Erythromycin (54%), Streptomycin (17%), and Amoxicillin (14%) were the most resistant antibiotics. Least resistance was observed to Ciprofloxacin (5%).

**Conclusion:**

Street foods of the selected towns were highly contaminated with various antibiotic-resistant organisms. Hence, the relevant authorities ought to ensure the proper handling of street food by enforcing safety measures. Additionally, they should initiate a widespread awareness campaign promoting the prudent use of antibiotics among both street food vendors and the broader population.

## Background

Street food is defined as the type of food and beverages that are prepared or sold by street vendors in public areas for human consumption, with or without the need for additional processing and preparation [1–3]. It is an important business that helps developing nations meet the food needs of the poor [4] and promote socioeconomic development by providing employment for many unemployed people [2, 5, 6]. Besides, they attract tourists in some Asian countries [5, 7].

Street foods are frequently prepared under unsanitary conditions and are displayed in public, exposing them to a high level of contamination [8–10]. In addition, vending sites worsen traffic congestion and cause inconvenience to pedestrians [7]. People who rely on such foods are frequently more concerned with their convenience than with issues of food safety and quality [11]. Street food vendors often fortify these products with various nutrients and food additives to improve their palatability and attract customers [1]. These contents, when combined with other environmental factors, may foster the growth of various pathogenic microorganisms [1, 6].

Lack of training on proper food handling and processing, poor personal hygiene of vendors, and unsanitary surroundings could all be contributing factors to the observed safety issues [2, 4], which often lead to the rise of various food-borne diseases that can be life-threatening [3]. The issue is most pronounced in countries with insufficient food safety laws, weak regulatory systems, limited financial resources for safer equipment, and food safety training [12].

Studies show that bacterial water and foodborne pathogens are the primary causes of over 250 known water and food-borne diseases and these pathogens, mainly affecting the gastrointestinal tract [13], pose a significant threat, causing fatal diarrheal illnesses, especially in developing nations [14]. Globally, diarrheal diseases remain a major health concern [14, 15], with 1.9 million reported deaths, predominantly in children under 5, with a higher burden in Sub-Saharan Africa [16].

Studies from Africa and other continents revealed that some microorganisms of public health concern detected in street foods include *Bacillus cereus, Escherichia coli, Clostridium perfringens, Proteus* spp., *Staphylococcus* spp., *Salmonella* spp., and others [2, 4, 9, 12, 17–23]. Infections caused by *Salmonella* and *Shigella* are among the major public health problems in tropical and subtropical regions of the world [17]. In Ethiopia, the carriage rate of *Salmonella* among food handlers ranges from 0.9 to 6.9%, whereas that of *Shigella* ranges from 0 to 5.9% [12].

The era of the 1940s was characterized by the introduction of antibiotics, revolutionizing modern medicine, and saving the lives of millions of people [24]. Thus, the health and life expectancy in humans, as well as animals, were greatly improved [25]. Antibiotic resistance (ABR) by pathogenic microorganisms has become a significant and increasing public health problem internationally [24, 26, 27]. The main driver is increased misuse of antimicrobial agents in humans and animals [28]. This problem is more significant in developing countries [29, 30]. Antimicrobial resistance (AMR) necessitates a worldwide ecological standpoint and is one of the most vital public health concerns, and there will be about annual death of 10 million people by 2050 and economic loss of 100 trillion USD due to AMR [31].

Food may be a vehicle for the transfer of antibiotic resistance (ABR) bacteria and antimicrobial resistance genes (ARGs) to humans anywhere along the food chain [25, 27]. At present, scientists in Ethiopia have documented the emergence of ABR in *Salmonella, Staphylococcus*, and *Shigella* species to common drugs like tetracycline, co-trimoxazole, chloramphenicol, ampicillin, streptomycin in varying degrees [4, 12, 26, 29].

In Ethiopia, few studies have documented the presence of various bacterial species with antibiotic susceptibility patterns (ASTs) in street food. In addition, the existing studies are of older dates, had methodological flaws in isolating bacteria from the food samples, and their scopes were limited to one city with small sample sizes. Hence, their value for decision- makers is limited. This study overcame some of the shortcomings of previous studies by covering a larger study area, using a larger sample, involving various street food staff and using standard microbial identification techniques to isolate bacteria. Therefore, this study aimed at in isolating the *S. aureus* and *Enterobacteriaceae* species and determining their antibiotic resistance patterns.

## Materials and methods

### Study design, setting, and period

Laboratory-based cross-sectional study design was used to determine the prevalence of Bacterial and antibiotic resistance patterns from street foods in Addis Ababa, Hawassa, Dire Dawa, and Jimma towns of Ethiopia from December 2022 to February 2023. The study took place in four purposively selected major urban cities in Ethiopia. Addis Ababa, Ethiopia’s capital city, Hawassa, the city of the Sidama regional states, is found 275 km to the south away from Addis Ababa. Jimma, the zonal town of Jimma Zonal Administration which is located 355 km to the southwest of Addis Ababa, and Dire Dawa, the city of Dire Dawa council administration, which is located 500 km to the east of Addis Ababa. These cities, marked by substantial population growth and industrial parks, employ numerous young individuals at lower wages. Due to high inflation and time constraints, residents often buy affordable street foods for basic physiological needs.

### Sample size and sampling technique

The number of street food vending stalls were determined using a single population proportion formula by considering 95% CI, a corresponding standard score of 1.96, and a margin of error (d) 0.05, 11.7% of *Staphylococcus aureus* prevalence on street food (P) [22]. Including a 10% non-response rate, the final sample size was 175 street food vending stalls.

The calculated sample size was proportionally distributed to the number of populations found in the selected four towns and sub-cities within each town since it was not possible to obtain the actual number of street food vending stalls found in each town. Based on the proportional allocation, 138, 15, 12, and 10 street food vending stalls from Addis Ababa, Hawassa, Dire Dawa, and Jimma cities were selected respectively. To select street food vending stalls, a stratified sampling technique was used by dividing the sub-city into five zones based on direction by following the main roads. Three randomly selected food items and drinking water samples were taken from a single vending stall, and a total of 525 food items and 175 water samples were collected to run the analysis.

### Collection and processing of samples

The entire study was divided into two steps. The first step was the isolation and identification of the bacteria from the sample by cultural, morphological, and biochemical tests, while the second step was the evaluation of antibiotic susceptibility patterns of the isolated bacteria.

### Collection of food samples

Three randomly selected food items (their nature and processing techniques described in Table 1) from a single vending stall were collected aseptically using three different sterile glove to take food items from each vending stall, kept in labeled sterile polyethylene plastic bags on ice, and immediately transported to Hawassa, Jimma, Haramaya Universities, medical laboratory departments, and Ethiopian Public Health Institute Laboratories, respectively. On arrival, samples were registered, and given a unique code. The samples were taken in the morning from 1:30AM to 3:30 AM where most consumers used them as a breakfast.

**Table 1:** Sampled street food items with their ingredients and description, in selected towns of Ethiopia, 2022

Street food name	Ingredients	Processing techniques
Sambussa/ samosa/	Wheat dough, salt, oil, chopped onions and red pepper, cooked rice, cooked lentils,	The dough prepared flatly and other ingredients rolled in triangle shape with it and roasted in deep frying oil.
Kokor/Ethiopian crunchy biscuit/	Wheat dough, salt, oil	Pre-determined dough made round/oval and put in boiled oil and fried
Ambasha/ Ethiopian wheat based flat bread/	Wheat dough, salt, baking powder, oil	The dough made flat on the hot plate and backed
Injera (flattened bread)	Teff (*Eragrostis tef)* dough	Fermented teff (*Eragrostis tef)* batter will be backed on hot plate and backed for 2–3 min
Omolicho	Ensete (*ensete ventricosum*), haricot been, chopped cabbage, onion, oil, salt, butter	All ingredients cooked step by step
Pasta	Wheat dough, chopped onions, meats, spices, oil, salt	Boiled pasta mixed with stew and other spices
Potato	Potato, salt, chili pepper, locally made spices (data/doko)	boiled Potato peeled and other ingredients will be added
Avocado	Bread, avocado, onion, salt	Avocado peeled and smashed then onion and salt added
Egg	Bread, egg,, oil, salt, onion, chili pepper	Egg roasted and other ingredients will be added
Bread	Wheat dough, salt, backing powder,	The dough will wrapped with the leaf of *ensete ventricosum* and backed
Bonbolino	Wheat dough, salt, sugar, oil	Wheat dough made circle open in the center and roasted in frying oil
Donut	Wheat dough, salt, melted sugar, oil	Wheat dough made circle open in the center and roasted in frying oil and pour the melted sugar on the top.

### Collection of water samples

Sterile 200 ml bottles containing 0.2 ml of 3% solution of sodium thiosulfate (to neutralize any chlorine) were used to collect the drinking water from the vending stalls and transported to the laboratory using cold chain [32, 33].

### Sample processing

#### Food sample process

The food samples were cut into smaller pieces using surgical blade and picked by sterile forceps and measured 25 g using digital balance aseptically. In a sterile 500 ml flask bottle, 25 g of ready-to-eat food was aseptically mixed with 225 ml of 0.1% buffered peptone water (OXOID, CM059) and homogenized by shaking for ten minutes with a Uni-jogger shaker to dislodge the bacteria from the food sample for the media. One ml of homogenate was transferred to a sterile test tube containing 9 ml of buffered peptone water to make 10^− 1^ to 10^− 5^ appropriate serial dilutions for the microbiological analysis and then incubated at a temperature of 37 °C for 24 h.

### Water sample process

Microbial water analysis was carried out using the membrane filtration technique, following the procedures described in American Public Health Association [34]. The absorbent pads were aseptically placed into petri dishes and saturated with Lauryl Sulfate Broth (HiMedia). A 200-mL water sample was filtered through a 0.45-µm membrane filter (HACH company), and the filter papers were put on the absorbent pad. The plates were then incubated at 37 °C for 24 h to detect total coliforms. After 24 h of incubation, colonies with a yellow color were counted and recorded. The risk was categorized according to World Health Organization classification as it is shown in Table 2 [13].

**Table 2:** Risk category of drinking water

*E. Coli* colonies forming Unit /100 ml	Risk category
0	Conformity for consumption
1–10	Low risk
11–100	Intermediate risk
101–1000	High risk
> 1000	Very high risk

### Bacterial isolation, identification, and enumeration

Using the pour plate technique, from the processed samples, 0.1 ml of the suspension was transferred to MacConkey (Mac) agar, Mannitol Salt Agar (MSA), and Salmonella Shigella Agar (SSA) (all were an Oxoid Ltd., UK) for Enterobacteriaceae, Staphylococcal [35], and Salmonella-Shigella counts, respectively. The plates were then incubated at 37^o^C for 24 h for bacterial growth [36].

The results of each plate having colonies were counted using a colony counter. Average counts obtained expressed as Colony Forming Units per gram of food (CFU/g) by multiplying the number of bacteria by the dilution factor. Selected isolates from Mac and SSA were then sub-cultured into nutrient agar and were incubated to make the sample refresh for different biochemical tests that were performed for the identification of gram negative bacteria. The biochemical tests used were Triple Sugar Iron, Lysine Decarboxylase, Simmons Citrate, Urea, and Sulfide Indole Motility. The inoculated biochemical tests were incubated for 18–24 h at 35–37 °C and checked for any color change. Gram stain and coagulase tests were undertaken for the subculture of the isolate that grew on MSA and nutrient agar. The gram stain is a differential stain which is used to differentiate bacteria into two groups Gram positive bacteria and Gram negative bacteria. The technique is based on the fact that Gram positive cell wall has a stronger attraction for crystal violet when iodine is applied and therefore retains the crystal violet and therefore will remain purple after decolorizing while Gram negative bacteria will be colorless after decolorizing with alcohol, counterstaining with Safranin will make them to appear pink.

In Catalase test the glass slide was held at an angle and few drops of 3% hydrogen peroxide were allowed to flow slowly over the culture. The emergence of bubbles from the organism was noted. The presence of bubble displayed a positive test indicating the presence of enzyme catalase. If no gas is produced, this is a negative reaction. All were conducted following the standards of the Clinical and Laboratory Standards Institute [37].

### Antibiotic susceptibility test

Antibiotic Sensitivity tests were carried out by using the Kirby–Bauer disc diffusion method using Mueller-Hinton Agar (MHA) following the recommendation of the Clinical and Laboratory Standards Institute [38]. The inoculum was prepared from three to five colonies of bacteria, transferred to a tube containing 5 ml of normal saline, and mixed gently to have a homogenous suspension. The absorbance was adjusted to 0.5 McFarland equivalents. The inoculum size (0.1 ml) of the bacterial suspension was then swabbed over the entire surface of Mueller-Hinton agar using a sterile cotton swab and the antibiotic discs were placed at the equidistance of the plate and incubated at 37 °C for 18 to 24 h. The diameter in millimeters of the zones of inhibition around each of the antimicrobial discs were measured and categorized as resistant, intermediate, and sensitive according to the company recommendations. The antibiotics used were Amoxicillin Clavulanic acid (AMC 30 𝜇g), Chloramphenicol, (C 30 𝜇g), Ciprofloxacin (CFX 5 𝜇g), Erythromycin (E 15 𝜇g), Streptomycin (S 10 𝜇g), and Sulphametoxazole-trimethoprim (SXT 25 𝜇g). These antibiotics were chosen based on their availability in the market and frequency of prescriptions in Ethiopia for the treatment of bacterial infections.

### Data quality

Aseptic technique was used throughout all sampling and handling procedures. Sterile polyethylene plastic bags to put the purchased food items and sterile bottles for sampled water were used, and transported under recommended conditions with ice cold and placed immediately in the refrigerator till analysis was started. To avoid unpredictable changes, samples were analyzed without delay under sterile biosafety cabinets. The preparation of each used medium was made according to the manufacturer’s instruction. The sterility of each medium was checked by incubating overnight at 37 °C. Media with any growth were discarded. Different reference bacterial strains were used for media and antimicrobial performance check.

### Statistical analysis

Data were coded, verified, and entered into the Statistical Package for the Social Sciences (SPSS) version 25.0 (SPSS Inc, Chicago, IL, USA) for analysis. Descriptive statistical tools were used to analyze frequency, percent, mean, and standard deviation. While Analytical statistics ANOVA was used to see the association between dependent (continues variables) and independent variables (type of food and towns) and presented as text and tables. *P-value* ≤ 0.05 was considered as statistically significant.

### Ethical clearance

Ethical clearance was obtained from the Jimma University Institute of Health Institutional Review Board. The Board provided ethical approval after reviewing both the protocol and consent forms. A support letter was given from the department of Environmental health, science and technology of Jimma University and given to towns’ administration offices and informed verbal consent was obtained from each study participants. Confidentiality was insured by collecting the data anonymously.

## Results

### Isolation of bacteria from street vended foods

Out of 525 food samples, 279 (53%) of the samples had at least one bacterial species. From 175 water samples, *E. coli* was detected in 95 (54.3%) of the samples. From the total of food and water samples, eleven bacterial species were identified. Among all tested food samples, 27.2% of them were found to be contaminated with *Staphylococcus aureus.* Its count in food items ranged from 5.4 × 10^3^ to 4.2 × 10^6^cfu/g. Among the samples, the highest number of *Staphylococcus aureus* was found in pasta, and the lowest in omolicho. The second most abundant organism was *E.coli* in 19% of the samples with the range 3.2 × 10^3^ to 8.4 × 10^5^ cfu/g. The highest prevalence of *E.coli* was detected in bonbolino (24.7%). the ANOVA result showed that *Shigella*, *Klebsiella*, and *Salmonella group A* species had statistical significant with the type of food items whereas, *Citrobactor*, *Shigella*, *Enterobacter*, and *Serratia* species had statistical significant among the cities as shown in Table 3.

**Table 3:** Microbial counts (CFU/g) of street food samples in selected towns of Ethiopia, 2022

Bacteria Name	Ranges	Mean	Standard deviation	Statistical significance	
				Between Food items	Between Towns
*Staphylococcus aureus*	5.4 × 10^3^ to 4.2 × 10^6^	3.2 × 10^5^	± 2.5 × 10^4^	0.920	0.729
*Citrobacter*	1.4 × 10^4^ to 1.2 × 10^6^	2.8 × 10^5^	± 4.4 × 10^5^	0.480	0.001*
*Shigella*	2.4 × 10^2^ to 2.5 × 10^6^	3.7 × 10^4^	± 1.5 × 10^5^	0.001*	0.001*
*E. coli*	3.2 × 10^3^ to 8.4 × 10^5^	1.9 × 10^5^	± 2.5 × 10^5^	0.918	0.302
*Klebsiella*	6.3 × 10^3^ to 7.6 × 10^5^	2.2 × 10^5^	± 2.7 × 10^5^	0.001*	0.331
*Providencia*	4.3 × 10^3^ to 6.8 × 10^5^	2.2 × 10^5^	± 3.0 × 10^5^	0.988	0.060
*Enterobacter*	8.6 × 10^3^ to 5.4 × 10^5^	1.5 × 10^5^	± 1.9 × 10^5^	0.944	0.010*
*Serratia*	4.6 × 10^3^ to 2.5 × 10^5^	5.4 × 10^4^	± 5.9 × 10^4^	0.952	0.032*
*Salmonella*	1.2 × 10^2^ to 2.5 × 10^4^	1.6 × 10^4^	± 2.5 × 10^4^	0.992	0.525
*Salmonella group A*	1.6 × 10^2^ to 6.7 × 10^3^	5.4 × 10^3^	± 1.0 × 10^4^	0.001*	0.070
*Shigella dysentery*	2.5 × 10^2^ to 4.6 × 10^3^	1.6 × 10^4^	± 2.5 × 10^4^	0.999	0.476

* Statistical significance at *p* < 0.05 in ANOVA analysis

Coagulase tests were conducted on the identified *Staphylococcus* organisms, and positive results were obtained in 17.5%, 19.4%, 35.3%, and 27.8% of Addis Ababa, Dire Dawa, Hawassa, and Jimma towns respectively. The highest rate of *Salmonella* contamination was found in omolicho (100%) and egg (50%), but it was not detected in pasta and injera. All types of the isolated bacterial species were identified in bonbolino, kokor, ambasha, and sambussa, with percentage of 27.6%, 21.1%, 18.6%, and 17.5% respectively. In omolicho, only *Salmonella* species were detected. *Staphylococcus aureus* was the most frequently isolated organism in almost all food items except in omolicho. Pasta and egg contain the largest proportion of *Staphylococcus aureus* bacterial species with the percentage of 67% and 50% respectively as shown in Table 4.

**Table 4:** Percentage of isolated bacteria from the sampled street foods in the selected towns of Ethiopia, 2022

Food type	Bacteria name											Total number
	E.coli	Kl.	S.A	Sh.	Sa.	Pro.	Ent.	Cit.	Ser.	Sa.A	Sh.Dy.	
Sambussa	20.4	4.1	32.7	12.2	4.1	2	2	2	8.2	4.1	8.2	49
Kokor	22	6.8	22	6.8	6.8	3.4	8.5	6.8	5.1	3.4	8.5	59
Bombolino	24.7	6.5	23.4	9.1	7.8	2.6	3.9	2.6	6.5	7.8	5.2	77
Ambasha	19.2	9.6	23.1	9.6	13.5	1.9	7.7	1.9	3.8	5.8	3.8	52
Potato	0	0	33.3	0	33.3	0	0	0	0	33.3	0	3
Pasta	0	0	66.7	0	0	0	0	0	0	0	33.3	3
Avocado	0	0	44.4	33.3	11.1	0	0	0	0	11.1	0	9
Egg	0	0	50	0	50	0	0	0	0	0	0	2
Bread	0	7.1	35.7	0	21.4	0	7.1	0	7.1	14.3	7.1	14
Omolicho	0	0	0	0	100	0	0	0	0	0	0	1
Injera	20	0	40	0	0	0	0	20	0	20	0	5
Donat	0	20	40	20	20	0	0	0	0	0	0	5
abundance of bacteria	53	18	76	26	27	6	14	9	15	18	17	279

Kl.: Klebsiella, S.A: staphylococcus, Sh.: Shigella species, Sa.: Salmonella species, Pro.: Providencia, Ent.: Enterobacter, Cit.: Citrobacter, Ser.: Serratia, Sa. A: Salmonella group A, Sh.Dy.: Shigella Dysentery

### Water sample result

**Fig. 1:**
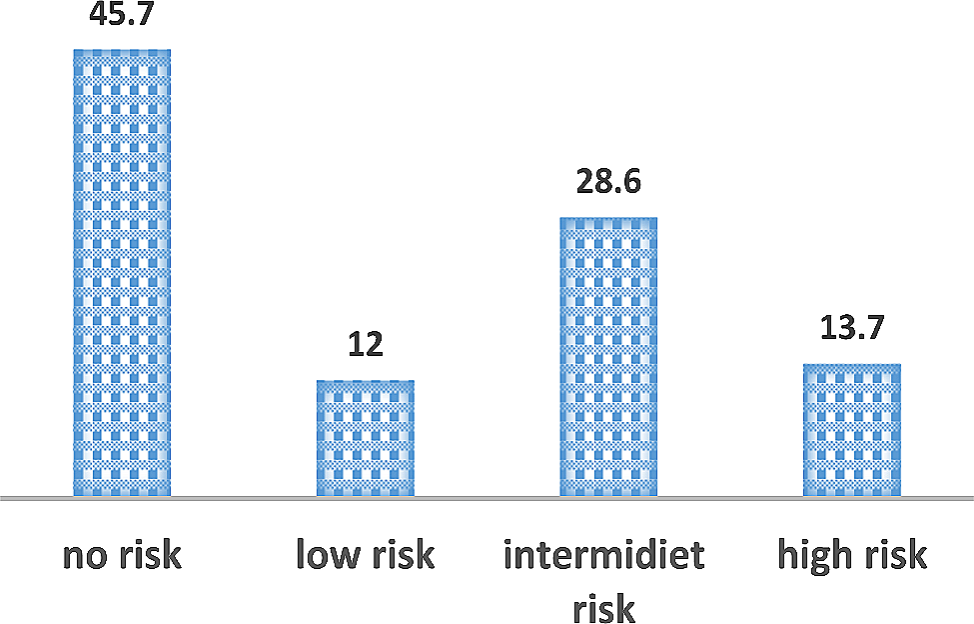
Percentage of Risk category of water sample from street food vending stalls in Ethiopia, 2022

Sampled water analysis showed that *E. coli* was detected in 95 (54.3%) samples. Among them, 21 (12%) had low risk while 24 (13.7%) were in the category of high risk as shown in Fig. 1.

### Antibiotic susceptibility test result

The isolated bacteria showed varying degrees of resistance to commonly used antibiotics. No organism showed resistance to all of the antibiotics tested. The three antibiotics with the highest resistance rates were erythromycin (78.6%), amoxicillin (36.4%), and streptomycin (36.4%).

The majority of *S. aureus* isolates (44.7%) were resistant to Erythromycin, followed by Streptomycin (17.1%). The isolates were, however, susceptible to Sulphametoxazole -trimethoprim (91.8%). *Salmonella* isolates were resistant to Erythromycin but sensitive to Ciprofloxacin, Amoxicillin, and Sulphametoxazole-trimethoprim. Half (50%) of *Shigella* species were resistant to Erythromycin, but sensitive to Sulphametoxazole-trimethoprim. The study documented that *E.coli* was most resistant to Erythromycin, but 100% sensitive to Ciprofloxacin. Ciprofloxacin was completely effective against *E. coli, Klebsiella, Shigella dysentery, Providencia, Enterobacter, Citerobacter, and Serratia*, as it is shown in Table 5.

**Table 5:** Percent of bacterial isolates resistance from street foods against commercial antibiotics in selected towns of Ethiopia, 2022

Bacteria	N	Antibiotics (Percentage of resistance)					
		AMC	C	CIP	E	S	SXT
*E. coli*	53	13.2	5.7	0	67.9	15.1	7.5
*Klebsiella*	18	16.7	11.1	0	72.2	22.2	16.7
*Staphylococcus aureus*	76	14.5	10.5	10.8	44.7	17.1	9.2
*Shigella*	26	19.2	11.5	7.7	50.0	15.4	3.8
*Salmonella*	27	7.4	14.8	7.4	29.6	18.5	7.4
*Providencia*	6	0	16.7	0	33.3	16.7	0
*Enterobacter*	14	28.6	0	0	78.6	21.4	14.3
*Citrobacter*	9	33.3	22.2	0	66.7	44.4	33.3
*Serratia*	15	7.7	0	0	66.7	6.7	6.7
*Salmonella group A*	18	13.3	11.1	16.7	50.0	22.2	16.7
*Shigella dysentery*	17	11.8	11.8	0	52.9	5.9	5.9
Total bacteria isolates	279	14.7	9.7	5.4	54.1	17.2	9.7

N = Total number of bacteria species, AMC Amoxicillin, C = Chloramphenicol, CIP = Ciprofloxacin, E = Erythromycin, S = Streptomycin, SXT = Sulphametoxazole–trimethoprim

## Discussion

The finding of this study revealed that, there is a dominance of *Staphylococcus aureus, E. coli, Salmonella*, and *Shigella* species in the sampled street foods. It is consistent with what other researchers have documented in different parts of the world [1–4, 6, 39–41]. Most likely, this is because of improper food handling and vendors’ poor hygienic practices [3, 39, 42].

Staphylococcal foodborne disease is one of the most common and frequent foodborne diseases in the world [6, 43]. The dominance of *Staphylococcus aureus* almost in all food items in this finding is similar to [4, 17, 21, 22]. The high prevalence could be associated with its resistance to heat [6] and the unsanitary behaviors like frequent touching of wounds, stroking hair scalp, burns, and dirty fomites of the food handlers [42]. The absence of *staphylococcus aureus* and other bacteria from omolicho might be due to the acidic nature of the food. As *Ensete ventricosum* ferments, its pH value decreases which inhibits bacterial growth [44]. The presence of the highest proportion of *Staphylococcus aureus* in pasta might be due to the high water activity [45], prior preparation, and inappropriate storage [40]. 

The other dominant bacterial species isolated was *E.coli*, which also is in line with previous studies [4, 21, 46]. This dominancy could be attributed to heat processing failure or post-processing fecal contamination due to poor hygienic practices of food handlers and poor water quality [2] This research confirms that 54% of water from street food stalls tested positive for *E. coli*. The presence of these bacteria is linked to factors such as inadequate water quality, use of dirty utensils, and contamination from fecally contaminated water, poor personal hygiene of vendors, and improper food storage [3, 39–41].

*Salmonellosis* is known for major food-borne zoonotic diseases transmitted through animal-derived foods [2]. The rate of *Salmonella* contamination in our finding was higher than that of the previously documented research in Gondar and Hawassa [4, 21]. The difference could be attributed to a procedural difference since they didn’t use enrichment media for the isolation of bacteria, but lower elsewhere [1, 17, 20]. Regional variations in animals and environmental reservoirs of *Salmonella* species as well as food preparation conditions can explain these variations [20]. The highest rate of *Salmonella* contamination in omolicho might be due to the ability of *Salmonella* to decrease proton extrusion and membrane proton conductance which enables the cell to be protected against acid stress [47]. The highest frequency in eggs might be due to the internal contamination of eggs prior to oviposition by the laying hen or immediate contamination of eggs following lay, due to contact with feces or fecally contaminated laying material [48]. High *Salmonella* load has also been correlated with the ability to survive on fingertips in contaminated vendors’ hands for long periods of time [49].

The detection of all species of bacteria from Bonbolino, Kokor, Ambasha, and Sambussa, is in concordance with research done in Jigjiga [22] and Hawassa [4]. This might be attributed to the prior processing of foods which exposes them to dust, flies, and other contaminants before they are available to the consumers.

Despite the advancement of many antibiotic discoveries, the development of antibiotic-resistant strains of bacteria has risen to the top of the list of global public health priorities [50]. It calls for concerted efforts to conserve the effectiveness of antibiotics for both preventing and treating infections, especially in developing countries [6, 21, 41, 50].

Our findings documented that the bacteria isolated from the ready-to-eat foods showed significant resistance to the tested antibiotics which is consistence with the findings of other researchers [2, 6, 41]. This might be due to the indiscriminate use of antibiotics in human health, animal husbandry, and agriculture [28, 50]. The resistance of *S. aureus* to Erythromycin was higher than that of a study done in Taiwan [51]. The susceptibility of this organism to Chloramphenicol, Ciprofloxacin, and Streptomycin was slightly lower than research documented by [4, 17, 41, 52], but higher than research done in Gondar and Haramaya, Ethiopia [29, 50]. This variation could be attributed to decrease in uptake of the antibiotics by the bacterial cells [45] and geographical differences.

*S. aureus*, *Salmonella*, and *Shigella* showed resistance to all tested drugs, suggesting challenges in treating related foodborne diseases. This resistance may lead to the survival and transfer of resistance traits, resulting in the development of multi-antibiotic resistant bacteria in the future [41, 53]. All of the bacteria tested in this study were highly susceptible to Ciprofloxacin, which is consistent with the findings of other researchers [4, 43].

Access to safe drinking water and sanitation services for all is a critical issue for public health and development [19]. *E. coli* detected in over 50% of water samples from street food stalls raises concerns about potential exposure to various harmful organisms, such as Salmonella and Shigella, not covered in this study [54]. Poor environmental conditions, and the use of old and dirty containers for water transportation are common factors contributing to the presence of *E. coli* [55, 56].

### Strength and limitations

The study’s strength was that it used a large number of samples, covered a wide area, and used a variety of street food items and water samples. Water quality has a wider scope that involves several parameters including fecal coliforms and physico-chemical parameters. However, this study focuses only on common bacteriological parameters (*E. coli*) that are considered in the determination of drinking water quality based on WHO standards. The other is, due to the cross-sectional nature of the study design, the finding was a one-time data analysis that may not indicate the seasonal variation of street food and water contamination. Thus repeated seasonal-based studies may be needed to investigate the actual gap. Besides, the research didn’t go further in identifying the bacteria at subspecies character using primer, RNA, and DNA techniques.

## Conclusion

This study discovered that 53% of the street food samples were contaminated with at least one species of bacteria. *E. coli* was found in 54.3% of the water samples. Eleven bacterial species were found in food and water samples. The study also found that *Staphylococcus* species were the most commonly detected organisms. All types of isolated bacterial species were identified from Bonbolino, Kokor, Ambasha, and Sambussa. Among all the antimicrobials tested against the isolates, no organisms displayed 100% resistance to the tested antimicrobial drugs. Erythromycin, Amoxicillin and Streptomycin were the three antibiotics with the highest resistance whereas; Ciprofloxacin was the most effective drug. Hence, the relevant authorities ought to ensure the proper handling of street food by enforcing safety measures. Additionally, they should initiate a widespread awareness campaign promoting the prudent use of antibiotics among both street food vendors and the broader population. Further studies should be conducted to explore the governance and control mechanisms of these street foods.

## Data Availability

Data supporting the finding of this research are available upon the reasonable request from the corresponding author.

## Abbreviations

ABR: Antibiotic resistance
ARGs: Antimicrobial resistance genes
ASTs: Antibiotic susceptibility patterns
CFU/g: Colony Forming Units per gram of food
Mac: MacConkey agar
MSA: Mannitol Salt Agar
MHA: Mueller-Hinton Agar
SSA: Salmonella Shigella Agar
Spps: Species
